# Troubleshooting Paracentesis Using POCUS

**DOI:** 10.24908/pocus.v8i2.16265

**Published:** 2023-11-27

**Authors:** Angelina Voronina, Nachelle Aurelien, Edward Bergin, Paula Roy-Burman

**Affiliations:** 1 Department of General Internal Medicine, Section of Hospital Medicine, Weill Cornell Medical Center; 2 Department of Pulmonary/Critical Care, New York-Presbyterian Queens; 3 Campbell County Memorial Hospital, Sidney Health Center

**Keywords:** Paracentesis, Procedures, Ascites, Complications

## Abstract

Paracentesis is a procedure routinely performed at the bedside in the evaluation and management of ascites. While point of care ultrasound (POCUS) assistance during paracentesis is known to reduce the risk of procedure-related complications, intraprocedural POCUS to overcome commonly occurring issues, such as obstructed flow through the centesis catheter, remain poorly described. In this report, we present two cases in which bowel adhered to the catheter during paracentesis. POCUS was used in an attempt to restore flow. Based on our literature review and procedural experience, we propose an algorithm to surmount this routinely encountered problem.

## Introduction

Ascites, or the pathologic accumulation of fluid within the abdominal cavity, can be the result of multiple processes. Cirrhosis is the most common etiology in the United States; left untreated, it portends a 60% risk of developing ascites within the first ten years of diagnosis 1, 2. Accumulation of ascites often necessitates fluid removal with paracentesis – a procedure of percutaneously inserting a catheter or hollow needle through the abdominal wall into the peritoneal space – for symptom relief and/or laboratory analysis. Medicare data demonstrate that from 1993 to 2008 the number of paracenteses performed in the United States have more than doubled from 64,371 to 149,699 3. Concomitantly, data from 2004 to 2012 demonstrate a 10% increase in the number of paracentesis procedures performed in the inpatient setting 4.

Paracentesis is a generally well-tolerated procedure. Adverse events are estimated at 1% and include infection, post-procedural leakage of ascitic fluid, abdominal wall hematoma, bowel perforation, and intraperitoneal hemorrhage 1, 5, 6, 7, 8, 9, 10, 11, 12, 13, 14, 15. There are additional intraprocedural concerns such as the aspiration of intestinal wall or omentum into the centesis catheter, or the placement of the catheter tip within the abdominal soft tissue. Though the frequency of these latter complications are not well described, they may result in unsuccessful paracentesis through disruption of ascites drainage 16.

Pre-procedural point of care ultrasound (POCUS) is known to minimize the risks of paracentesis by identifying a safe procedural site, and is widely acknowledged as the standard of care 5, 7, 11, 12, 13, 14, 15, 17. However, there is a paucity of literature which considers the role of intraprocedural POCUS in troubleshooting poor or interrupted ascitic drainage. Here, we present two cases of failed peritoneal drainage and the techniques utilized to restore flow through the catheter. We analyze our experience and posit how direct visualization by POCUS may improve the success of paracentesis drainage.

## Case 1

**Figure 1:**
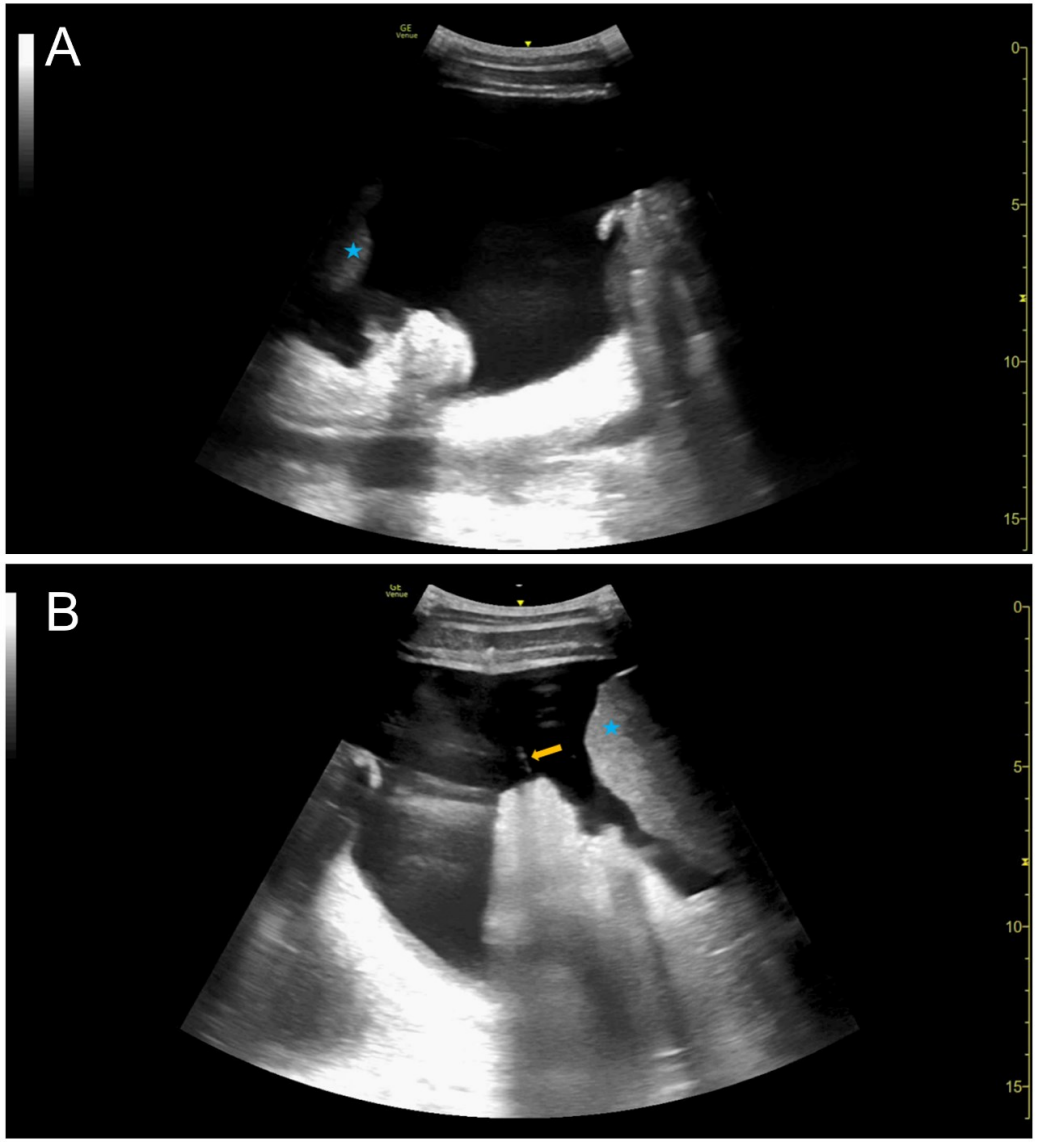
23-year-old man with metastatic cancer of unknown origin complicated by ascites. A) Pre-procedure POCUS exam demonstrating a large pocket of ascites in the right lower quadrant of the patient described in Case 1. [Asterisk = liver] B) Intraprocedural POCUS demonstrating paracentesis catheter attached to omentum/bowel. Note that the ultrasound probe was inadvertently flipped in this image. [Arrow = catheter; asterisk = liver]

A 23-year-old man with metastatic cancer of unknown origin complicated by ascites requiring repeated intraperitoneal drainage presented to the hospital with abdominal pain, distension, and early satiety. Our inpatient procedure service was consulted for therapeutic paracentesis. POCUS was used to identify a safe pocket in the right lower quadrant, approximately 10 cm in depth (Figure 1a). Ultrasound-assisted paracentesis was performed according to standard protocol adopted by our local institution. Eight-hundred milliliters of amber-colored fluid collected into an evacuated container before drainage abruptly ceased. The operators attempted to restore flow through the intraperitoneal catheter by discontinuing vacuum suction (i.e., closing the three-way stopcock on the tubing system), rotating the catheter by 180° in clockwise and counterclockwise directions, and attempting manual aspiration via one-way syringe. These techniques resulted in minimal drainage (<20 mL). The aforementioned steps were repeated with gentle retraction of the catheter in one-centimeter increments without success. At this juncture, POCUS was employed under sterile conditions and demonstrated persistent ascites and attachment of bowel to the paracentesis catheter (Figure 1b).

## Case 2

**Figure 2:**
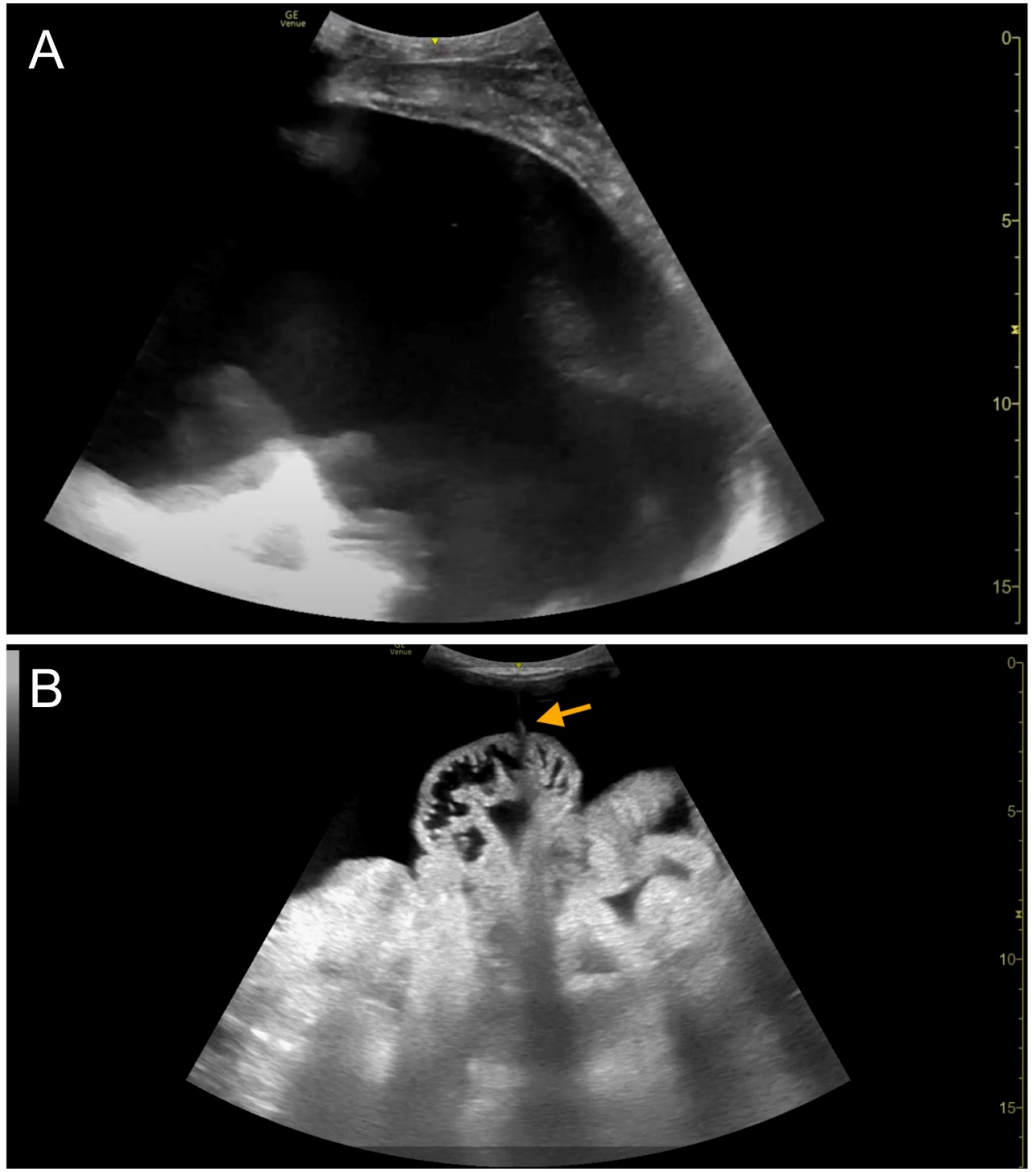
68-year-old woman with decompensated alcoholic cirrhosis. A) Pre-procedural POCUS exam demonstrating large volume ascites in the left lower quadrant of the patient described in Case 2. B) Intraprocedural POCUS exam demonstrating attachment of omentum/bowel to the paracentesis catheter. [Arrow = catheter]

A 68-year-old woman with decompensated alcoholic cirrhosis presented to the hospital with altered mental status and acute kidney injury in the setting of medication noncompliance. She was determined to have hepatic encephalopathy and poor renal perfusion secondary to abdominal compartment syndrome. Our inpatient procedure service was consulted to perform a large-volume paracentesis. Following standard protocol, ultrasound-assisted paracentesis was performed in the left lower quadrant after identifying free-flowing ascites approximately 10 cm in depth (Figure 2a). After initial drainage of 1.1 L of clear, yellow fluid into evacuated containers, flow through the paracentesis catheter abruptly terminated. Given ongoing physical exam findings of a tense, distended abdomen, and pre-procedural insonation of significant peritoneal fluid, obstruction of flow through the catheter was suspected. POCUS performed with sterile technique demonstrated attachment of bowel with retraction of the catheter (Figure 2b; Supplemental Video S1).

## Discussion

In both cases, a common technical challenge was encountered: interruption of ascites drainage by bowel/omentum. Here, we discuss how intraprocedural POCUS examination was relevant to our attempts to restore flow through the centesis catheter. Our intention is to provide proceduralists of all skill levels a practical approach to this commonly encountered problem. We begin with a summary of the literature available on tactics to address disrupted catheter flow, and ultimately offer a troubleshooting algorithm for proceduralists.

While numerous sources describe the paracentesis technique and the use of ultrasound to decrease the risk of adverse events, formal literature on procedural troubleshooting is scant. The most systematic approach to changes in flow through the intraperitoneal catheter was found in a procedure manual developed by the Canadian Internal Medicine Ultrasound (CIMUS) group 2. Recommended troubleshooting techniques included inspection of the procedure set-up for leaks or loss of vacuum, adjustment of the catheter to address flow obstruction, and adjustment of the patient’s position to address changes in ascites volume with drainage. All of the sources we identified provided recommendations to address obstructed flow through a catheter, which is suggestive of the relative frequency of this complication. The use of POCUS to assess the procedure area, untwisting the catheter, and flushing the catheter with sterile saline were all proposed as techniques to issues with catheter flow 18, 19. (A complete list of references and recommendations can be found in Table 1.) 

**Table 1:** Troubleshooting techniques. Table summarizing real-time procedural adjustments in the setting of failed peritoneal drainage.

-	**RECOMMENDED TECHNIQUE**
**Verify procedure setup**	
Desy et al., 2021 2	Check for leaks or loss of vacuum in the procedure set up
**Confirm ongoing ascites**	
Wheeler, 2019 18	Percuss abdomen
Wheeler, 2019 18 Killu et al., 2017 20	Assess the procedure area with ultrasound
**Adjust for poor drainage**	
Desy et al., 2021 2 Killu et al., 2017 20 Glauser et al., 2008 21	Reposition the patient (e.g., rotate patient, elevate head of bed, etc.)
**Adjust for obstructed catheter**	
Desy et al., 2021 2 Wheeler, 2019 18 Glauser et al., 2008 21	Manipulate catheter (e.g., re-angulate, rotate, and/or retract)
Jeong et al., 2015 19 Killu et al., 2017 20 Glauser et al., 2008 21	Release suction at the catheter tip (e.g., close and reopen stopcock)
Wheeler, 2019 18 Jeong et al., 2015 20	Flush catheter

We compared these techniques with our experience. In Case 1, after troubleshooting techniques were employed, the problem was verified by direct visualization using ultrasound (Supplemental Video S2). Sterile saline was flushed incrementally in an attempt to release the bowel from the catheter and visualized entering the peritoneal space. The bowel, however, remained attached. The procedure was terminated by withdrawing the catheter while applying gentle pressure at the insertion site. The patient did not report any discomfort. On repeat insonation, the omentum and bowel were visualized as free-floating. Paracentesis was performed from a different site, and 3 L of amber-colored peritoneal fluid was successfully removed without complication.

In Case 2, troubleshooting techniques were attempted after confirmation of obstructed catheter flow. Flow was initially restored by first closing the three-way stopcock towards the patient to release any suction, and then gently rotating the catheter 180° clockwise and counterclockwise to free the bowel from the catheter side port. Approximately 150 mL of ascitic fluid was drained before flow was again obstructed. The aforementioned steps were repeated and the procedure was resumed utilizing a 60 mL syringe for manual aspiration. Another 100 mL of fluid was aspirated before flow was again disrupted by the bowel. A saline flush was then attached to the three-way stopcock, and the device opened toward the patient. After rapid and repeated infusion of 10 mL saline (Supplemental Video S3), an additional 1 L of ascitic fluid was removed before flow abruptly terminated. Given repeated obstruction and persistent large volume ascites, the procedure was discontinued. Paracentesis was reattempted in the right lower quadrant with removal of 2.3 L of clear, yellow fluid. The patient tolerated both procedures well and did not experience any complications.

Though intraprocedural POCUS was employed in both cases, the timing of its use differed. Whereas ultrasound was introduced at the outset in Case 2, ultrasound was utilized after several troubleshooting techniques were first attempted in Case 1. We speculate that the earlier use of intraprocedural POCUS contributed to the total amount of ascites successfully aspirated in Case 2. 

POCUS has the potential to rapidly elucidate the etiology of disrupted flow by confirming the presence of ongoing ascites and visualizing catheter position. The techniques used to restore flow through the catheter can be monitored and adjusted prior to reattempting aspiration. Premature aspiration while the catheter is still blocked has the potential to worsen the obstruction such that subsequent troubleshooting efforts are unsuccessful, necessitating a repeat procedure and further time spent at the bedside.

**Figure 3:**
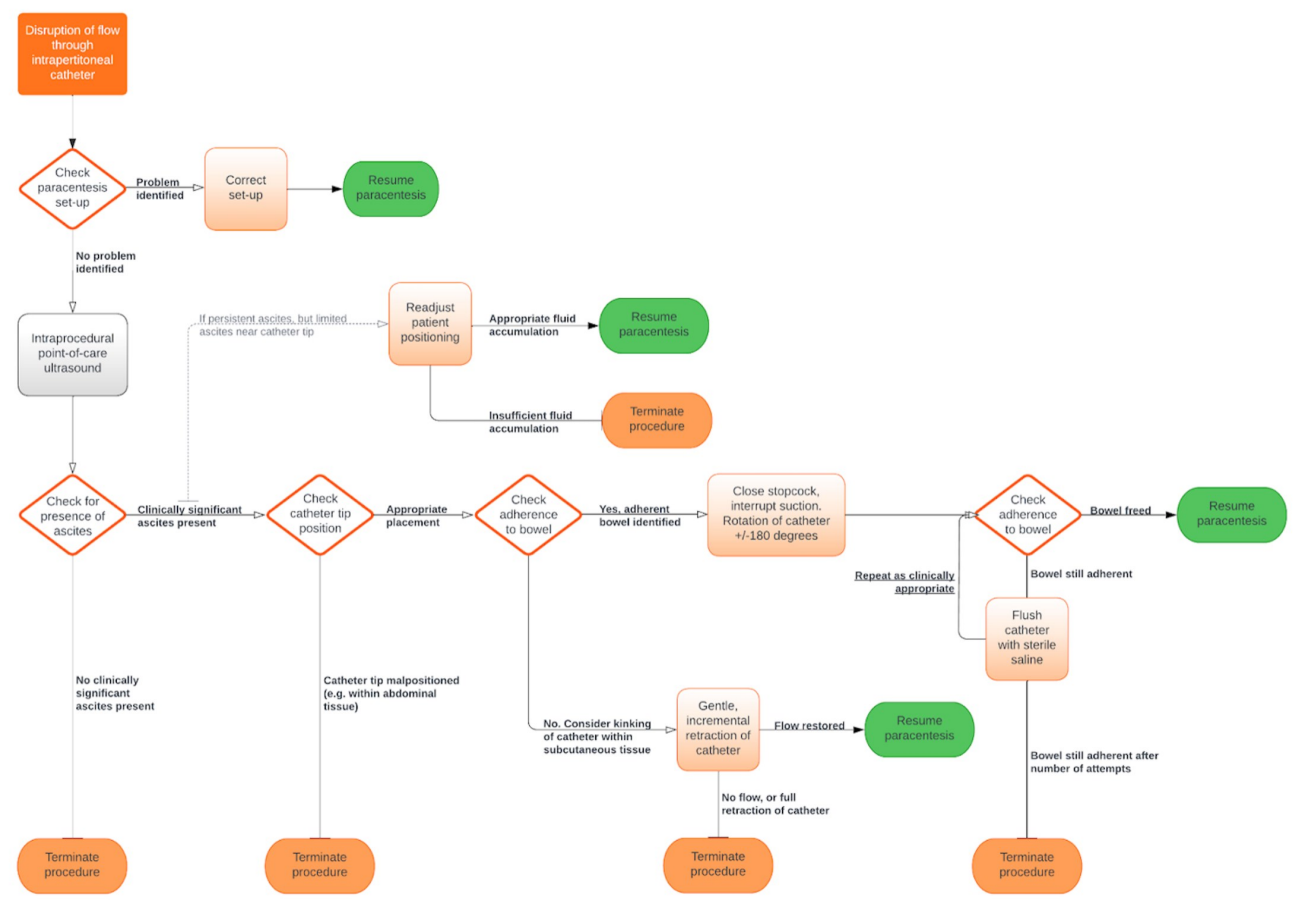
Proposed paracentesis troubleshooting algorithm. Systematic approach to identifying the etiology of obstructed peritoneal drainage and improve catheter flow during paracentesis.

Considering the existing literature and our case experiences, we propose an algorithm to systematically address issues with catheter flow (Figure 3). In the first step, the system of tubing and connections is assessed. This includes verifying that attachments are appropriate (e.g., stopcock valves turned in the correct direction), and without external kinks or air leaks (e.g., loss of vacuum from an evacuated container). Intraprocedural ultrasound is then incorporated to confirm presence of ongoing ascites and identify catheter position. If the catheter is obstructed by intraperitoneal structure such as bowel, a series of techniques (i.e., releasing suction, twisting the catheter, flushing with saline, etc.) can be attempted. The success of each effort is monitored by ultrasound. Switching from vacuum to manual aspiration may be helpful in controlling the degree of negative pressure applied to the system and prevent repeated bowel attachment.

When attempting to flush the catheter port with fluid, we find that rapid infusion yields the most success. We favor a small 10-20 mL syringe as larger syringes have more resistance. Syringes should be attached to the stopcock at the base of the centesis catheter to limit the distance the fluid must travel. It may be helpful to rotate the catheter at the time of flushing. Bowel may also be freed from the catheter with gentle retraction. Direct visualization of the peritoneal space using POCUS can determine the location of the catheter tip in relation to the volume of ascites present. This information can guide the proceduralist to retract the catheter more liberally than the one centimeter increments often recommended by expert opinion. If after repeated attempts, the bowel fails to detach from the catheter, the procedure should be terminated and reattempted at a different site, if needed. The optimal number of attempts and the amount of fluid to be tolerated is yet undetermined.

To implement this algorithm, proceduralists will need materials in addition to routine paracentesis supplies (Table 2). Most importantly, we recommend having an ultrasound on hand with a sterile probe cover and conducting medium to maintain a sterile procedure field. In an optimized scenario, an assistant would be available to operate the ultrasound machine while the proceduralist remains sterile. 

**Table 2:** Modified paracentesis procedure checklist. Revised procedure checklist to accommodate for intraprocedural ultrasound. Additional elements are denoted by an asterisk (*).

• Paracentesis Kit
• Fluid collection system (e.g., evacuated containers and vials for laboratory specimens)
• Personal protective equipment (e.g., sterile gown, gloves, and mask)
• Skin antiseptic (e.g., chlorhexidine or iodine)
• Ultrasound machine and gel
• Sterile probe cover*
• Sterile ultrasound gel*
• Sterile saline*
• Various sized sterile syringes (e.g., 10-35 mL)*

## Conclusion

The rapid determination of obstructed flow through a paracentesis catheter has the potential to increase successful paracentesis, decrease the number of reattempts, and save valuable time of the proceduralist at the bedside. We propose an algorithm which uniquely incorporates intraprocedural POCUS and serial reassessment in hopes to systematically approach this commonly encountered problem. Formalized studies are necessary to evaluate success of such an algorithm and its component recommendations.

## Ethics statement

The authors of this manuscript have all agreed to authorship. They have read and approved the manuscript, and given consent for submission and subsequent publication. All authors have been actively involved in the substantial work leading to the paper and will take public responsibility for its content.

Informed consent was obtained per hospital protocol for the two cases; one of the patients is deceased.

## Disclosures

The authors report no relevant disclosures related to this work.

## Supplementary Material

 Video S1 Bowel moving toward peritoneum with retraction of the catheter. The catheter is not well visualized.

Video S2 Catheter is visualized at the end of the clip. Arrow indicating location of the catheter.

Video S3 Saline flush infusion with visualization of attachment of the bowel on the left side of the screen. Catheter is not well visualized.
